# In vivo Effects of *Salicornia herbacea* and *Calystegia soldanella* Extracts for Memory Improvement

**DOI:** 10.4014/jmb.2312.12045

**Published:** 2024-03-13

**Authors:** Jiun Sang, Seeta Poudel, Youngseok Lee

**Affiliations:** Department of Bio and Fermentation Convergence Technology, Kookmin University, Seoul 02707, Republic of Korea

**Keywords:** Memory enhancement, *Salicornia herbacea*, *Calystegia soldanella*, *Drosophila melanogaster*, Parkinson’s disease model, taste-associative memory

## Abstract

The global elderly population, aged 65 and over, reached approximately 10% in 2020, and this proportion is expected to continue rising. Therefore, the prevalence of neurodegenerative diseases such as Parkinson’s disease (PD), which are characterized by declining memory capabilities, is anticipated to increase. In a previous study, we successfully restored the diminished memory capabilities in a fruit fly model of PD by administering an omija extract. To identify functional ingredients that can enhance memory akin to the effects of the omija extract, we conducted screenings by administering halophyte extracts to the PD model. Halophytes are plants that thrive in high-salt environments, and given Korea’s geographic proximity to the sea on three sides, it serves as an optimal hub for the utilization of these plants. Upon examining the effects of the oral administration of 12 halophyte extracts, *Salicornia herbacea* and *Calystegia soldanella* emerged as potential candidates for ameliorating memory loss in PD model flies. Moreover, our findings suggested that *C. soldanella*, but not *S. herbacea*, can mitigate oxidative stress in *DJ-1β* mutants.

## Introduction

In recent years, the global elderly population aged 65 and over has experienced a significant upsurge, reaching approximately 10% in 2020, with projections indicating a continued upward trajectory, especially in developed countries [1]. This demographic shift has been accompanied by a corresponding rise in the prevalence of various medical issues, particularly neurodegenerative conditions such as Parkinson’s disease (PD), Alzheimer’s disease, Huntington’s disease, and others. A prevailing hallmark of these neurodegenerative diseases is the gradual decline of memory capabilities, which significantly impacts the quality of life of affected individuals.

Our previous research has focused on combating the decline of memory functions within a fruit fly model of PD, and promising results were achieved through the administration of an omija extract [2]. Building on these findings, our current research has sought to identify additional functional ingredients with potential memory-enhancing effects. To achieve this, we conducted comprehensive screenings, evaluating the effects of halophyte extracts on loss-of-function mutations in *DJ-1β* (hereinafter *DJ-1β^ex54^*), which is among the PD models. *Drosophila melanogaster* possesses two *DJ-1* orthologs, namely *DJ-1α* and *DJ-1β*. *DJ-1α* is predominantly localized in the testes, whereas *DJ-1β* exhibits a broader expression profile across various tissues [3, 4]. This expression pattern of *Drosophila* *DJ-1β* parallels the distribution observed in mammalian *DJ-1* [5]. *DJ-1β^ex54^* has been documented to lead to heightened sensitivity to oxidative stress, elevated susceptibility to locomotor dysfunction, impaired taste-associative memory, decreased number of dopaminergic neurons, and a reduced survival rate [2-4, 6-9]. Halophytes, widely known for their adaptation to high-salt environments [10], have garnered substantial interest due to their potential therapeutic properties. Due to its unique geographical position, being surrounded by the sea on three sides, Korea has emerged as an ideal environment for the exploration and utilization of these plant species. Here, we selected 12 halophytes with potential memory-enhancing properties in the context of human health and well-being based on their nutritional value and potential therapeutic properties (Table 1). For example, some of the possible functions and health benefits associated with *Salicornia herbacea* include cardiovascular health, anti-inflammatory properties, antioxidant activity, and potential hypolipidemic effects [11]. *Calystegia soldanella* is believed to participate in antioxidant support, digestive health, skin health, and diuretic effects [12]. *Aster tripolium* is rich in nutrients and possesses potential antioxidant and anti-inflammatory properties, in addition to promoting cardiovascular and digestive health and exerting immunomodulatory effects [13]. *Carex scabrifolia* has been traditionally used for various therapeutic purposes due to its digestive health-promoting, diuretic, anti-inflammatory, and antioxidant properties [14]. Despite limited knowledge of the specific benefits of *Plantago major* var. japonica for. yezomaritima, broader knowledge of the *Plantago major* species suggests its potential applicability in traditional medicine, nutrition, wound healing, and antimicrobial support, making it an area of interest for further investigation and exploration [15-17]. However, the potential health benefits of *Lathyrus japonica* var. aleuticus, *Lysimachia mauritiana*, *Peucedanum japonicum*, *Suaeda asparagoides*, *Suaeda maritima*, *Angelica japonica*, and *Spergularia marina* have remained largely unexplored. Here, we identified *Salicornia herbacea* and *Calystegia soldanella* as uniquely promising candidates for counteracting memory loss in PD. Furthermore, our findings suggest that *C. soldanella* could alleviate oxidative stress within the *DJ-1β* mutant condition, further underscoring its potential therapeutic significance.

## Materials and Methods

### Fly Stocks

*w^1118^*(BDSC 5905) was used as a control. The *DJ-1β^ex54^* fly line was generously provided by Dr. J. Chung [4].

### Chemical Reagents and Halophyte Extracts

Sucrose (CAS No. 57-50-1) and caffeine (CAS No. 58-08-2) were purchased from Sigma-Aldrich (USA). Halophyte extracts of *Salicornia herbacea* (PB2617.1), *Calystegia soldanella* (PB4285.4), *Aster tripolium* (PB4826.1), *Carex scabrifolia* (PB1838.1), *Lathyrus japonica* var. aleuticus (PB3492.1), *Lysimachia mauritiana* (PB4143.1), *Peucedanum japonicum* (PB4051.2), *Suaeda asparagoides* (PB2619.2), *Suaeda maritima* (PB2620.3), *Plantago major* for. yezomaritima (Koidz.) Ohwi (PB4564.2), *Angelica japonica* (PB4043.6), and *Spergularia marina* (PB2646.1) were purchased from the Korea Research Institute of Bioscience and Biotechnology (Republic of Korea). The halophyte extracts were extracted using 99.99% methyl alcohol (HPLC grade).

### Taste-Associative Memory Assay

The taste-associative memory assays were performed as described in a previous study [2]. 7–8-day-old male flies were allowed to feed on cornmeal with or without 0.1% halophyte extracts. After 7 days of feeding, the flies were subjected to a 12–18 h fasting period. The flies were then anesthetized with ice, after which they were affixed to glass slides using nail polish. Approximately 10 to 15 flies were employed in each assay. Next, the flies were allowed to recover in a 60% humidified incubator at 25°C for a minimum of 2 h. Our experiment was structured into three distinct phases. The initial phase consisted of a pretest in which a 500 mM sucrose solution was applied to the flies’ legs. Flies exhibiting a positive proboscis extension in response to this stimulus were selected for further testing. This was followed by the training phase, during which 500 mM of sucrose was once again applied to the flies’ legs along with the simultaneous exposure to an aversive taste (50 mM caffeine) applied to the labellum. Each fly underwent this training process 15 times, and data from the training phase were categorized into three sets of five trials. Upon completion of the training phase, the flies were subjected to 500 mM sucrose stimuli on their legs at various time intervals (0, 5, 15, 30, 45, and 60 min), and their proboscis extension responses were recorded. Memory tests were conducted on both control and *DJ-1β^ex54^* flies after being fed either normal food or food supplemented with 0.1% of each halophyte extract for 7 days.

### Survival Assay under ROS Stress

Survival assays were performed as described previously [18] with slight modifications. 7–8-day-old male and female flies (15 male and 15 female) were allowed to feed on cornmeal with or without 0.1% halophyte extracts. After 7 days of feeding, the flies were transferred into vials containing 5% sucrose and 1% agar with or without 1% H_2_O_2_ at 25°C. The dead flies were counted at 12-h intervals, after which they were transferred to a new vial.

### Sleep Assay

The sleep assays were performed as described previously [19] with slight modifications. *Drosophila* activity monitor systems (DAMs) [20] were employed to automatically track fly activity in 1-min intervals. Activity was logged each time a fly crossed an infrared beam positioned in the middle of the recording tube, which the flies are incapable of perceiving. Flies exhibiting immobility for more than 5 min were classified as being in a state of sleep. The experimental setup included a 12-h light/12-h dark cycle at 25°C, with the aforementioned photoperiod serving as the basis for distinguishing between daytime and nighttime. 7–8-day-old male flies were transferred to vessels containing cornmeal with or without 0.1% halophyte extracts. After 7 days of feeding, the flies were transferred into glass tubes containing 1% sucrose in 1% agarose at one end and capped with a cotton ball on the other end. DAMs, equipped with glass tubes, were placed in the incubator 18 h before initiating the recording experiment. Data were collected and averaged over a 2-day period. Latency, defined as the time required to fall asleep after the lights were turned off, was then measured. Bout length was defined as the duration of uninterrupted sleep time, whereas bout number was defined as the number of uninterrupted sleep episodes. Sleep time, bout length, maximum bout length, bout number, and activity number were individually calculated for both daytime and nighttime. Thirty-two flies were analyzed for each experiment.

### RT-PCR Analysis

Gene expression levels were quantified using reverse transcription quantitative PCR. Thirty 7–8-day-old male flies were allowed to feed on cornmeal with or without 0.1% halophyte extracts. After 7 days of feeding, total RNA was extracted from their entire bodies using TRIzol reagent (Invitrogen), followed by cDNA synthesis using AMV transcriptase (Promega). For RT-PCR, specific primer pairs were employed for the *dlp* gene (forward primer: 5’-CAC ATC CCC AGT GGA ATC AC-3’; reverse primer: 5’-TGC CAA CAT TGA TCT GCT TC-3’), with *actin* (forward primer: 5’-CAC CGG TAT CGT TCT GGA CT-3’; reverse primer: 5’-GCG GTG GTG GTG AAA GAG TA-3’) serving as the reference gene.

The expression of each gene was quantified using ImageJ (ver. 1.53t; National Institutes of Health, USA). The region of interest containing the DNA was selected and converted to a grey-scale image, after which the image was quantified with ImageJ.

### CAFÉ Assay

CAFÉ assays were performed as described previously [21] with slight modifications. Groups of three males and three females, aged 7–8 days, were transferred to empty vials. Notably, the cornmeal-based food was presented in a liquid form using Standard Glass Capillaries (Cat. No. IB150F-3, World Precision Instruments, USA) to enable precise measurement of ingestion. Every 100 ml of liquid cornmeal food formulation consisted of 85.8 ml of tap water, 6 g of cornmeal, 1.5 g of brewer’s yeast, and 5.8 g of molasses, which were thoroughly mixed with heating at a high temperature. Additionally, variants of cornmeal food enriched with 0.1% *S. herbacea* or *C. soldanella* were prepared. Six standard glass capillaries, each containing cornmeal food with or without the halophyte additives, were placed in a single vial, and the ingestion volume was measured once daily for 7 days. Evaporation control measurements were also taken in the absence of flies at the same time, and the subtracted volume was calculated (*n* = 4). The reported ingestion volumes represent the average of the measurements from the sum of the six standard glass capillaries.

### Statistical Analysis

The error bars on the graph indicate the standard error of the means (SEMs); pairwise comparisons were conducted using Student’s *t*-test. All statistical analyses were performed using Origin Pro 8 for Windows (ver. 8.0932; Origin Lab Corporation, USA). Single-factor ANOVA coupled with Scheffe’s post hoc test was conducted to compare multiple datasets. Statistical significance was denoted using asterisks (*p < 0.05, **p < 0.01). Survival curves were examined using Kaplan–Meier analysis. Log-rank values were calculated by comparing the outcomes of each experiment.

## Results and Discussion

### Impaired Taste-Associative Memory in *DJ-1β^ex54^* and Restoration of Memory via Pral Administration of Halophyte Extracts

**Fig. 1:**
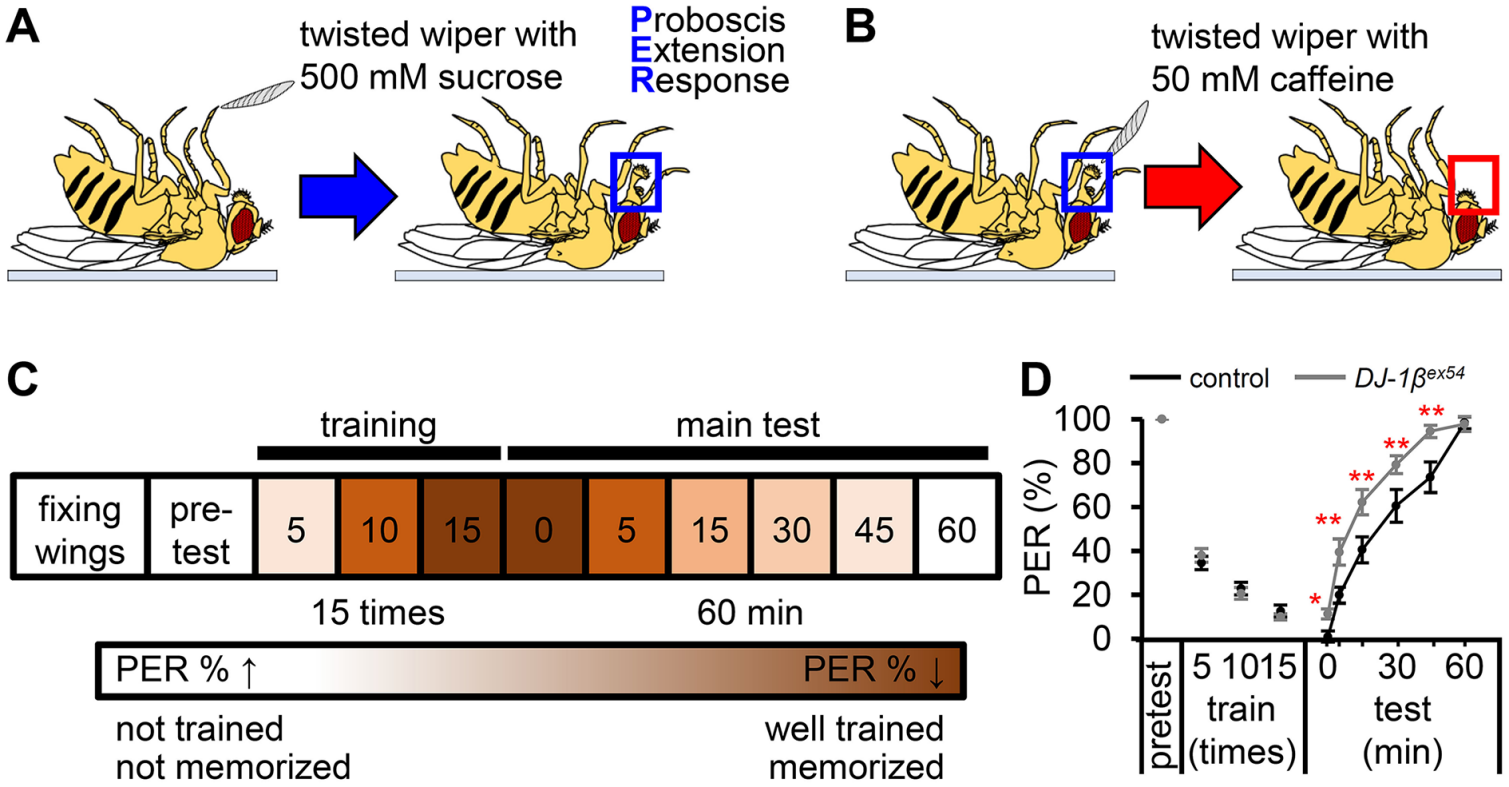
Taste-associative memory assay based on the proboscis extension response. (**A**) Schematic illustration showing the proboscis extension response (**PER**) when the forelegs of a fruit fly are stimulated with a wiper soaked in 500 mM sucrose, an attractive chemical. (**B**) Schematic illustration showing the disappearance of the PER when fruit flies with their proboscis stretched are presented with 50 mM caffeine, an aversive chemical. (**C**) Schematic diagram illustrating a tasteassociative memory assay for training and memory tests. (**D**) Both control and *DJ-1β^ex54^* mutant flies were trained with 500 mM sucrose and 50 mM caffeine for 15 trials and were subsequently tested with 500 mM sucrose at various time intervals (5, 15, 30, 45, and 60 min) to assess the acquisition of taste memory (*n* = 6). Error bars indicate the standard error of the means (SEMs). Pairwise comparisons were conducted using Student's *t*-test. Statistical significance is denoted by asterisks (*p < 0.05, **p < 0.01).

In our aging society, the rise in memory loss poses a significant public health concern with limited effective solutions. Therefore, the use of the associative taste memory assay as a screening tool for dietary health supplements geared towards enhancing memory represents a promising avenue [2]. The screen involved subjecting *D. melanogaster* to the taste-associative memory assay, utilizing the proboscis extension response (PER) as a measure of memory retention. This testing method, similar to Pavlovian theory, assesses learning ability and taste-associative memory by determining whether the subject can behave conditionally. In the taste associative memory assay, the use of caffeine, a chemical substance that elicits an aversive response in fruit flies, and sucrose, a chemical substance preferred by fruit flies, serves as the foundation for establishing a conditioned response in the flies. To prepare the flies for the assay, they were allowed to fast for 15–18 h to ensure their receptiveness to the chemical stimuli. Additionally, the flies were immobilized using nail polish, enabling precise exposure to the solutions during the experiment (Fig. 1A). During the experimental procedure, the flies’ legs were exposed to a 500 mM sucrose solution, prompting them to adopt a feeding posture, a behavior characterized by the extension of their proboscis (Fig. 1A). Conversely, exposure to compounds such as caffeine, which the fruit flies generally find displeasing, resulted in the cessation of the PER (Fig. 1B). We first selected the flies that exhibited a 100% response rate to sucrose, as indicated in the pretest phase (Fig. 1C). Next, we conducted a series of 15 training trials, each comprising the presentation of 500 mM sucrose to the leg, immediately followed by the application of 50 mM caffeine to the proboscis. Notably, each training session consisted of five trials, and the average data from three sessions are illustrated in Fig. 1C and 1D.

Deficits in learning and memory constitute crucial components of neurodegenerative diseases [22]. Neurotransmitters such as dopamine and serotonin play pivotal roles in the intricate processes of learning and memory. Parkinson’s disease (PD) is characterized by the degeneration of dopaminergic neurons [23], suggesting potential impairments in taste-associated memory within PD model flies [2]. In *D. melanogaster*, dopaminergic neurons are involved in various activities including locomotion, sleep, appetite, and aversive memory [24-26]. Flies that had formed an association between sucrose and the aversive taste of caffeine displayed a lower PER rate to sucrose immediately after training, with this association gradually diminishing over time. Notably, the *DJ-1β^ex54^* mutants exhibited increased PER responses compared to the control group during the test phase, indicating a deficiency in their capacity to form associative memories (Fig. 1D).

**Fig. 2:**
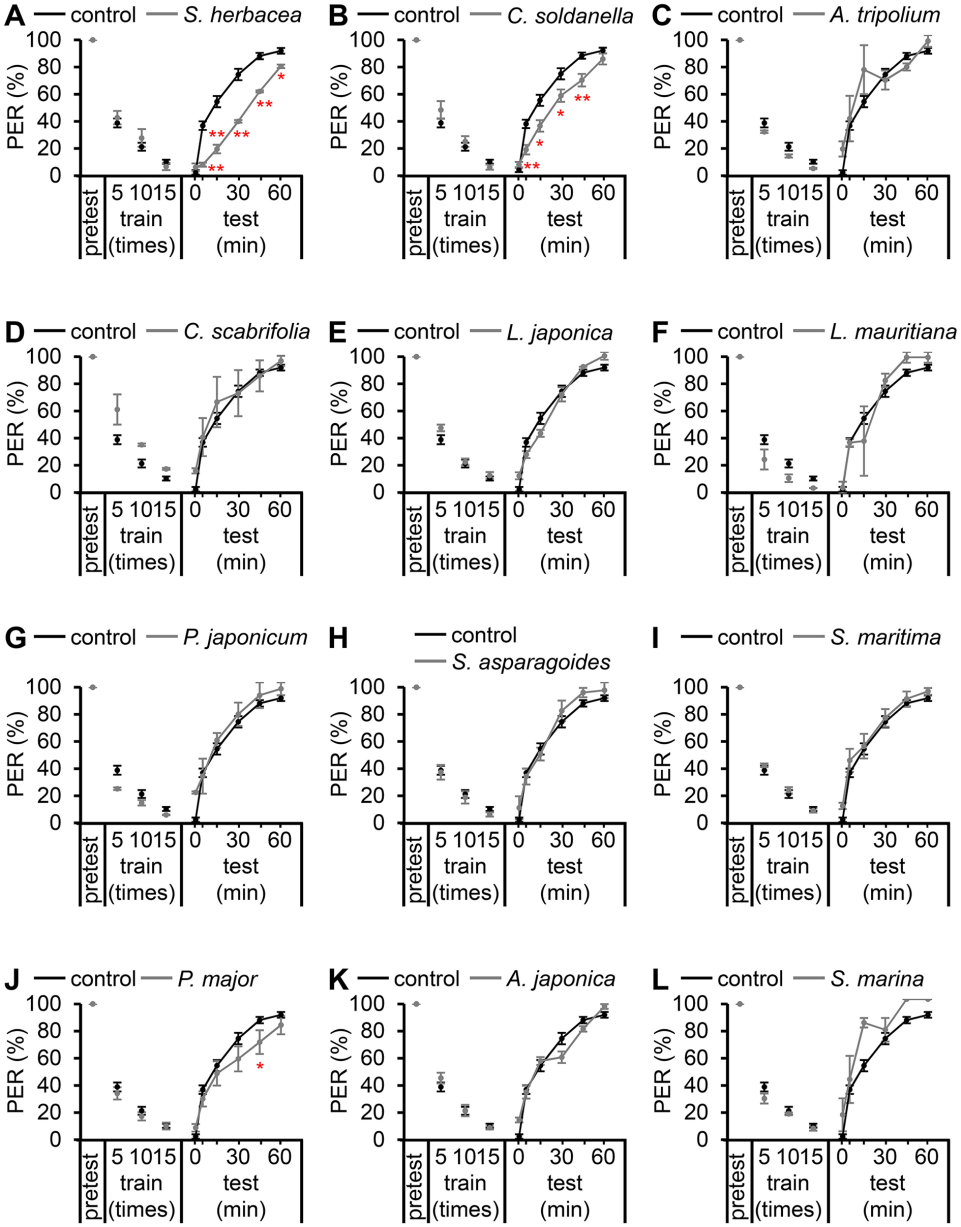
Enhancing taste-associative memory in a PD model: exploring the impact of halophyte consumption. (**A**) Effects of 0.1% *Salicornia herbacea* extract consumption on taste-associative memory in *DJ-1β^ex54^* mutants (*n* = 4). (**B**) Effects of 0.1% *Calystegia soldanella* extract consumption on taste-associative memory in *DJ-1β^ex54^* mutants (*n* = 7). (**C**) Effects of 0.1% *Aster tripolium* extract consumption on taste-associative memory in *DJ-1β^ex54^* mutants (*n* = 2). (**D**) Effects of 0.1% *Carex scabrifolia* extract consumption on taste-associative memory in *DJ-1β^ex54^* mutants (*n* = 3). (**E**) Effects of 0.1% *Lathyrus japonica* var. aleuticus extract consumption on taste-associative memory in *DJ-1β^ex54^* mutants (*n* = 2). (**F**) Effects of 0.1% *Lysimachia mauritiana* extract consumption on taste-associative memory in *DJ-1β^ex54^* mutants (*n* = 2). (**G**) Effects of 0.1% *Peucedanum japonicum* extract consumption on taste-associative memory in *DJ-1β^ex54^* mutants (*n* = 2). (**H**) Effects of 0.1% *Suaeda asparagoides* extract consumption on taste-associative memory in *DJ-1β^ex54^* mutants (*n* = 4). (**I**) Effects of 0.1% *Suaeda maritima* extract consumption on taste-associative memory in *DJ-1β^ex54^* mutants (*n* = 6). (**J**) Effects of 0.1% *Plantago major* for. yezomaritima (Koidz.) Ohwi extract consumption on taste-associative memory in *DJ-1β^ex54^* mutants (*n* = 9). (**K**) Effects of 0.1% *Angelica japonica* extract consumption on taste-associative memory in *DJ-1β^ex54^* mutants (*n* = 6). (**L**) Effects of 0.1% *Spergularia marina* extract consumption on taste-associative memory in *DJ-1β^ex54^* mutants (*n* = 2). The error bars indicate SEMs. Pairwise comparisons were conducted using Student’s *t*-test. Statistical significance is denoted by asterisks (*p < 0.05, **p < 0.01).

To this end, we conducted a small-scale screen encompassing 12 natural compounds to identify supplements with potential memory-enhancing properties. Each halophyte was orally administered to the flies at a concentration of 0.1% in cornmeal for 7 days to assess any discernible impact on the flies’ ability to form and retain associative memories. Out of the 12 groups that ingested these extracts, two groups exhibited a decreased PER rate compared to the group that consumed standard food. Specifically, the consumption of *Salicornia herbacea* (Fig. 2A) and *Calystegia soldanella* (Fig. 2B) yielded these outcomes. Notably, the intake of *S. herbacea* improved taste-associative learning and memory and consistently displayed significantly lower PER values at 5, 15, 30, 45, and 60 min (Fig. 2A). Similarly, the consumption of *C. soldanella* exhibited relatively reduced PER values at 5, 15, 30, and 45 min (Fig. 2B). On the other hand, no improvements in memory deficits were observed in the other 10 groups (Fig. 2C—2L). This suggests that the diminished taste-associative memory function resulting from the removal of *DJ-1β* was rectified by the consumption of *S. herbacea* and *C. soldanella*, respectively. Nevertheless, the therapeutic effects of the other compounds, potentially at different concentrations, cannot be ruled out.

### *S. herbacea* and *C. soldanella* May Enhance Memory Retrieval in Wild-Type Flies

**Fig. 3:**
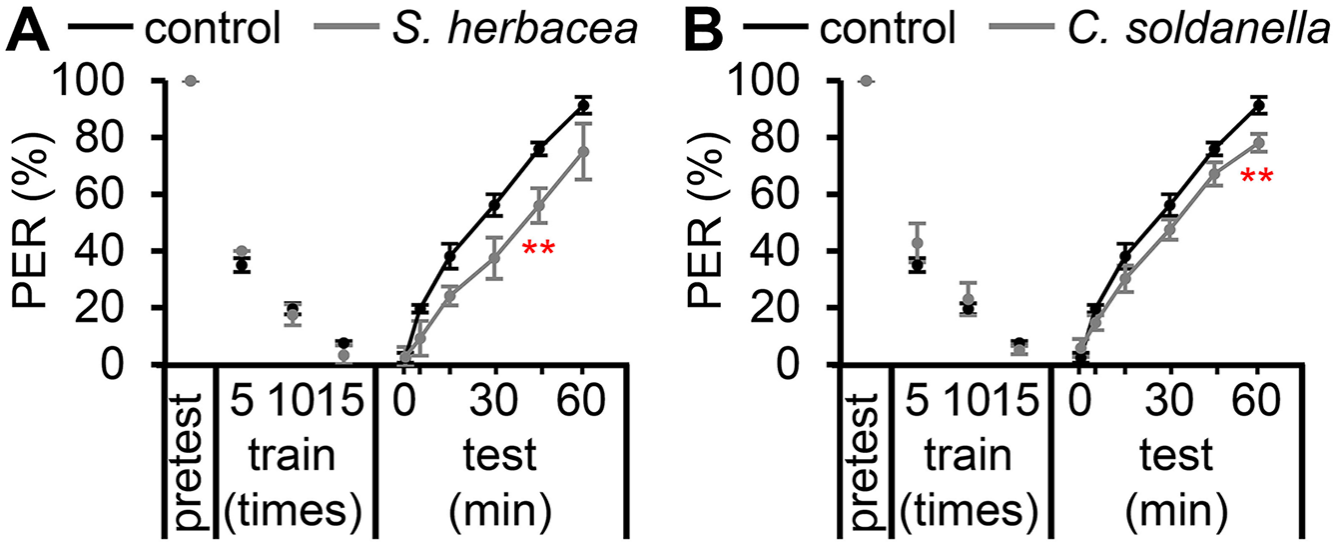
Effects of oral administration of *S. herbacea* and *C. soldanella* extracts on taste-associative memory in wild-type flies. (**A**) Effects of 0.1% *S. herbacea* extract consumption on taste-associative memory in control flies (*n* = 4). (**B**) Effects of 0.1% *C. soldanella* extract consumption on taste-associative memory in control flies (*n* = 9). The error bars indicate SEMs. Pairwise comparisons were conducted using Student’s *t*-test. Statistical significance is denoted by asterisks (**p < 0.01).

Next, we sought to examine whether the administration of *S. herbacea* and *C. soldanella* had any adverse or beneficial impacts on wild-type flies (Fig. 3A and 3B). Our findings revealed that both extracts had a noticeable improvement in memory, albeit with limited statistical significance at specific time intervals. However, it can be inferred that neither of the extracts had any detrimental impact on the memory of the control group. It is important to note a clear limitation in drawing conclusions about the beneficial effects of *S. herbacea* and *C. soldanella* on wild-type flies, as the observed effects of the extracts in the wild-type group were considerably less pronounced than those in the PD model flies.

### *C. soldanella* Downregulates *dlp* Expression, thereby Participating in the Mitigation of Oxidative Stress within the Context of *DJ-1β* Mutation

**Fig. 4:**
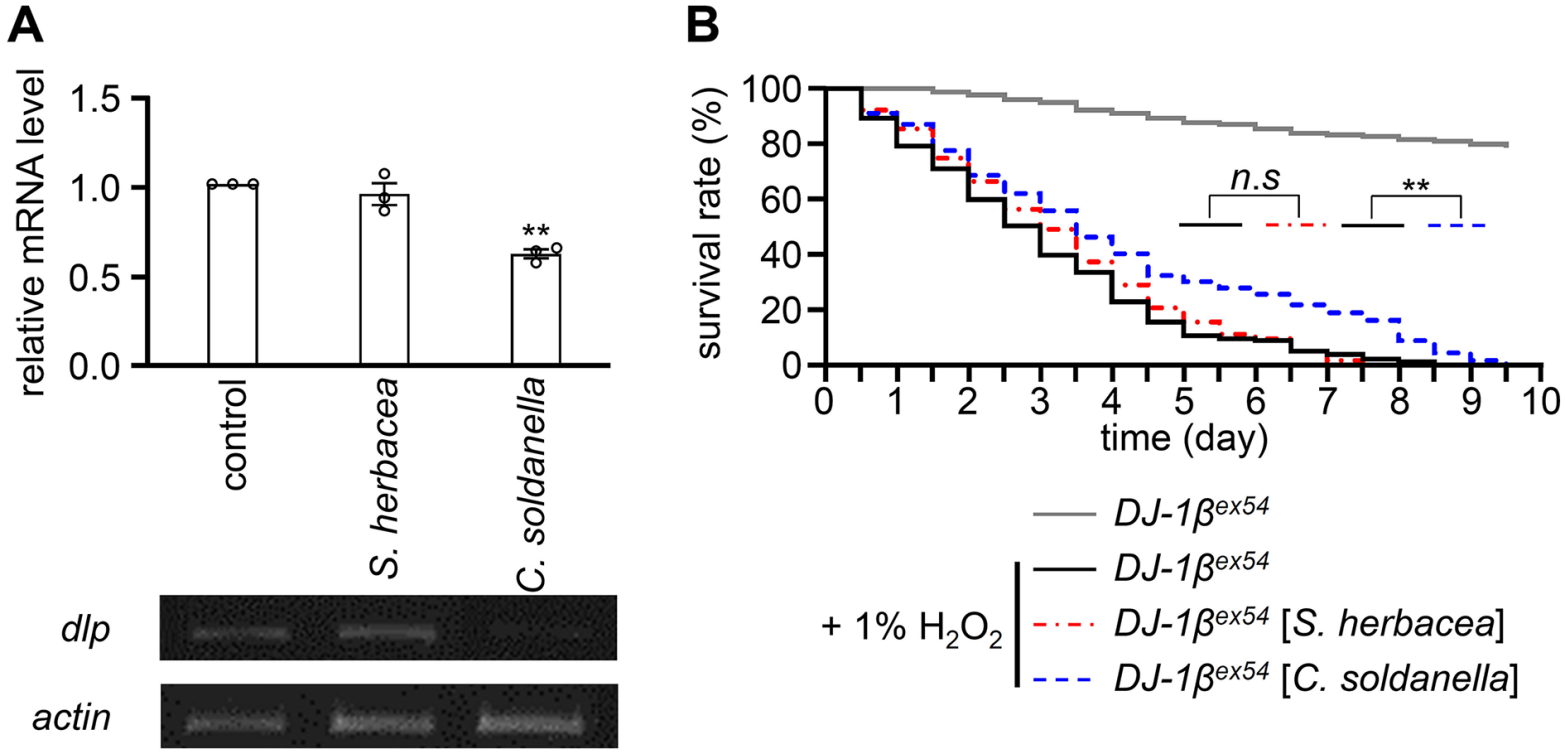
Exploration of the antioxidative effects of *S. herbacea* and *C. soldanella*. 7–8-day-old *DJ-1β^ex54^* flies were fed cornmeal with or without 0.1% of the indicated halophyte extracts, after which mRNA levels and survival rates were measured. (**A**) Measurement of *dlp* and actin gene expression levels in *DJ-1β^ex54^* flies fed 0.1% *S. herbacea* or *C. soldanella* extracts, respectively (*n* = 4). (**B**) Effects of 0.1% *S. herbacea* or *C. soldanella* extract consumption on lifespan under oxidative stress conditions (1% H_2_O_2_) in *DJ-1β^ex54^* mutant flies (*n* = 8; 240 flies).

The role of *DJ-1β* in impeding oxidative stress-induced neuronal apoptosis involves the regulation of *dlp* gene expression and its subcellular distribution, suggesting a potential link between mutations in *DJ-1β* and the loss of DA neurons in PD. In *Drosophila*, DLP operates both as a pro-apoptotic gene and an activator of the JNK pathway, similar to its mammalian equivalent Daxx [9]. Previous research has demonstrated the upregulation of Daxx in reaction to oxidative stress and UV exposure [9]. Particularly, exposure to H_2_O_2_ and UV radiation has been found to increase the expression of DLP. Given the significant role of DLP as a vital mediator of heightened sensitivity to oxidative stress in *DJ-1β* mutants, an investigation was initiated to examine *dlp* expression after the administration of two extracts (Fig. 4A). Initially, we quantified the effects of cornmeal with or without 0.1% *S. herbacea* and *C. soldanella* over a 7-day period. The calculated ingestion amounts (Test food - Evaporation) were determined to be 13.65 ± 0.75 ng for 0.1% *S. herbacea* and 13.38 ± 0.58 ng for 0.1% *C. soldanella* per fly throughout the 7-day experiment. In the experiment, the group that ingested *C. soldanella* exhibited a reduction in *dlp* mRNA levels in contrast to the group that consumed regular food. On the other hand, the group that consumed *S. herbacea* did not show any notable difference (Fig. 4A). Next, the impact of oxidative stress on the lifespan of *Drosophila* was investigated in groups fed 5% sucrose with 1% H_2_O_2_ after a diet with or without 0.1% of the aforementioned halophytes (Fig. 4B). A notable extension in lifespan was observed in the group fed with *C. soldanella*. Flies that had ingested 1% H_2_O_2_ to induce oxidative stress had a median survival of 3.2 ± 0.3 days, with all individuals dying by 7.5 days. Flies that ingested *S. herbacea* also had a median survival of 2.8 ±0.2 day, with all individuals dying by 7.5 days. In contrast, the group that ingested *C. soldanella* had a median survival of 3.5 ± 0.3 days, and all individuals died by 9.5 days, showing statistical significance when compared to the group that ingested 1% H_2_O_2_. The findings from this study indicate that *C. soldanella* may enhance taste-associative learning and memory through the reduction of oxidative stress. In contrast, *S. herbacea* appears to enhance memory through an unknown mechanism. Further investigations are needed to elucidate the specific mechanisms underlying the memory-enhancing effects of *S. herbacea*, providing valuable insights for future studies.

### *S. herbacea* and *C. soldanella* Supplementation Does Not Enhance Sleep Quality in *DJ-1β* Mutants

**Fig. 5:**
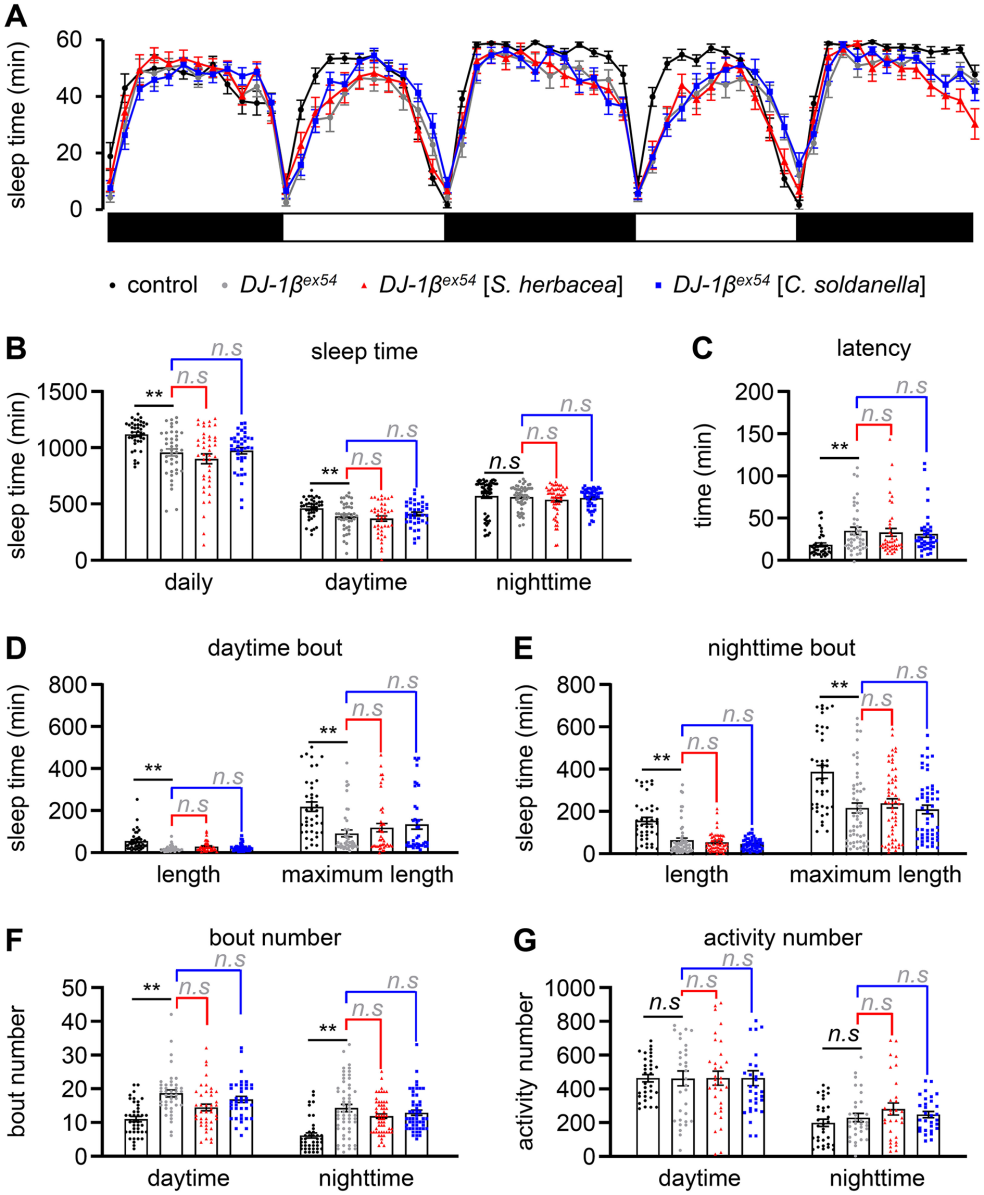
Analyzing sleep quality analysis: impact of *S. herbacea* and *C. soldanella* supplementation. 7–8-day-old flies were fed cornmeal with or without 0.1% of the indicated halophyte extracts, after which sleep assays (32 flies) were conducted with 1% sucrose and 1% agar. (**A**) Sleep pattern of control (*w^1118^*) flies and *DJ-1β^ex54^* mutant flies with or without oral administration of the indicated extracts. The white and dark bars indicate day and night, respectively. (**B**) Total average sleep amount at daytime and nighttime. (**C**) Sleep latency at night. (**D**) Average bout length and maximum bout length at daytime. (**E**) Average bout length and maximum bout length at nighttime. (**F**) Average bout number at daytime and nighttime. (**G**) Average activity number at daytime and nighttime. Single-factor ANOVA coupled with Scheffe’s post hoc test was conducted to compare multiple datasets. The black asterisks (**p < 0.01) indicate statistical significance compared to the control. The effect of plant extract supplementation is indicated in gray (n.s means non-significance).

Sleep disturbances are common in individuals with PD [27]. PD can affect various aspects of sleep, leading to disruptions in sleep patterns. Some common sleep-related issues in PD include insomnia and fragmented sleep. Managing Parkinson’s symptoms and implementing good sleep hygiene practices can contribute to improving sleep quality for individuals with PD. Despite lacking eyelids, fruit flies, like many other animals, exhibit diurnal behavior and undergo sleep episodes. Medications can have different effects on sleep. Some medications may cause drowsiness or fatigue as side effects, while others may interfere with sleep patterns. Stimulant medications, for example, may interfere with sleep and cause insomnia. On the other hand, certain medications, such as sedatives or antihistamines, may have a drowsiness-inducing effect. Some medications may also influence the sleep architecture, altering the balance of different sleep stages. *D. melanogaster* exhibits sleep-like states, and studying their sleep patterns has provided key insights into the genetic and molecular basis of sleep [28]. *D. melanogaster* harbors various genes linked to disruptions in these rhythms, which have also been observed in certain PD patients [29]. Notably, dopaminergic neurons in fruit flies exhibit similar activity levels during wakefulness and REM sleep, and mutations in genes such as *DJ-1β* can lead to the degeneration of dopaminergic neurons [30, 31]. First, we conducted a sleep analysis with control and *DJ-1β^ex54^* flies (Fig. 5A). The total sleep durations for the control and *DJ-1β^ex54^* flies were 1118.6 ± 19.1 min and 957.5 ± 31.5 min, respectively (Fig. 5B). Similarly, the daytime sleep duration was 458.5 ± 15.0 min for the wild-type and 386.5 ±19.1 min for *DJ-1β^ex54^*. Although both total and daytime sleep durations were relatively lower in *DJ-1β^ex54^* compared to the control, there was no statistically significant difference in nighttime sleep duration. Latency was 20.2 ± 2.2 min for the control and 36.8 ± 4.3 min for *DJ-1β^ex54^*, meaning that *DJ-1β^ex54^* took longer to fall asleep compared to the control (Fig. 5C). Both bout length and maximum bout length in *DJ-1β^ex54^* showed decreased values compared to the control during both day and night (Fig. 5D and 5E). The bout number during both day and night for the control was 10.8 ± 0.8 and 6.0 ± 0.8, respectively, whereas for *DJ-1β^ex54^*, it was 18.5 ±1.0 and 14.1 ±1.1, indicating an increased bout number compared to the control (Fig. 5F). There was no difference in basal activity number between the examined genotypes without any stimuli (Fig. 5G). *DJ-1β^ex54^* exhibited a reduction in daytime sleep duration, indicating a deterioration in sleep quality when considering the increased latency, decreased bout length and maximum bout length, and increased bout number.

In the context of sleep and PD in fruit flies, it is worth noting that supplementation with *S. herbacea* and *C. soldanella* did not yield improvements in sleep parameters such as sleep time, latency, sleep bout length, or sleep bout number in PD (Fig. 5). The observed findings suggest that the regulation of sleep in PD may not exhibit a direct and clear correlation with memory function.

Collectively, our findings suggest that the efficacy of *S. herbacea* and *C. soldanella* in ameliorating memory loss may be attributed to distinct mechanisms. Moreover, these treatments did not appear to have any appreciable effects on sleep. Physiologically active compounds were individually extracted from the two halophyte sources. *S. herbacea* yielded tungtungmadic acid, quercetin 3-O-glucoside, and isorhamnetin 3-O-glucoside [11]. Notably, tungtungmadic acid has been found to protect plasmid DNA from strand breakage induced by Fe^3+^-nitrilotriacetic acid-hydrogen peroxide [32]. Isorhamnetin 3-O-glucoside has shown inhibitory effects on adipogenesis by suppressing pro-adipogenic transcription factors in 3T3-L1 preadipocytes, potentially preventing obesity by regulating blood lipid profiles and adipose tissue weight [33]. Kaempferol-like compounds have been extracted from *C. soldanella*. However, the specific physiological effects associated with this extract remain unreported [34]. The identified ingredients, particularly those from *S. herbacea*, hold the potential to enhance learning and memory in PD model flies. Nevertheless, additional studies are needed to unveil the active ingredients and mechanisms underlying the memory-enhancing effects of these extracts.

The potential involvement of *C. soldanella* in triggering an antioxidative effect via the *DJ-1β*-DLP pathway provides a promising direction for upcoming research efforts. This may include the isolation and characterization of compounds, mechanistic studies to investigate downstream effects of antioxidative modulation, assessments of bioavailability and pharmacokinetics, in vivo studies, clinical trials, exploration of synergistic effects between compounds in *C. soldanella* and *S. herbacea*, examination of long-term effects, and formulation development. Exploring these topics in future studies may provide key insights into the therapeutic properties of *C. soldanella*-and *S. herbacea*-derived compounds, potentially contributing to the development of novel treatments for memory-related disorders.

## Figures and Tables

**Table 1:** List of the examined halophytes and their functions.

Halophytes	Functions	Ref.
*Salicornia herbacea*	Antioxidant activity, cardiovascular health, anti-inflammatory properties, and potential hypolipidemic effects	[11]
*Calystegia soldanella*	Antioxidant activity, digestive health, skin health, and diuretic effects	[12]
*Aster tripolium*	Antioxidant activity, nutritional richness, anti-inflammatory properties, cardiovascular health support, digestive health benefits, and immunomodulatory effects	[13]
*Carex scabrifolia*	Antioxidant activity, digestive health, diuretic properties, and antiinflammatory effects	[14]
*Plantago major* var. japonica for. yezomaritima	Nutritional richness, wound healing, and antimicrobial support	[15-17]
*Lathyrus japonica* var. aleuticus	Unknown	
*Lysimachia mauritiana*	Unknown	
*Peucedanum japonicum*	Unknown	
*Suaeda asparagoides*	Unknown	
*Suaeda maritima*	Unknown	
*Angelica japonica*	Unknown	
*Spergularia marina*	Unknown	
